# Survival Analysis of Lymphoepithelioma-Like Carcinoma of the Urinary Bladder and the Effect of Surgical Treatment Modalities on Prognosis

**DOI:** 10.3389/fsurg.2021.706537

**Published:** 2021-10-07

**Authors:** Jie Wu, Jian-Zhong Shou, Yu-Chen Wang

**Affiliations:** ^1^Department of Urology, National Cancer Center/National Clinical Research Center for Cancer/Cancer Hospital, Chinese Academy of Medical Sciences and Peking Union Medical College, Beijing, China; ^2^Chinese Academy of Sciences (CAS) Key Laboratory of Nutrition, Metabolism and Food Safety, Shanghai Institute of Nutrition and Health, University of Chinese Academy of Sciences, Chinese Academy of Sciences, Beijing, China

**Keywords:** urinary bladder neoplasm, lymphoepithelioma-like, radical cystectomy, prognosis, SEER

## Abstract

**Purpose:** This study aimed to investigate the prognostic factors of patients with lymphoepithelioma-like carcinoma of the urinary bladder (LELCB) and explore the value of surgical treatment.

**Methods:** Data of patients with LELCB were extracted from the Surveillance, Epidemiology, and End Results (SEER) database. The multivariate analysis was performed using the stepwise Cox proportional hazards regression model and conditional inference tree method to identify significant prognosticators of overall survival (OS) from the parameters such as age, gender, lymph node involvement, tumor extent, radiation, chemotherapy, and surgery type. Literature review (LR) was performed, and eligible cases were used to validate prognostic classification using the Kaplan–Meier method with log-rank tests.

**Results:** Sixty patients with a median age of 69.5 years were identified from the SEER database and 91 patients through LR. The Cox analysis identified age, gender, lymph node involvement, and surgical approach as independent prognosticators of OS. Based on the nomogram scores, patients were stratified into three prognostic groups: (I) patients younger than 70 years; (II) patients older than 70 years, who received bladder-sparing therapy (BST); and (III) patients older than 70 years undergoing radical cystectomy (RC). Patients in group II had the worst outcomes in terms of OS compared with patients in groups I and III (*p* < 0.001 and *p* = 0.03, respectively). A similar survival pattern was found in the LR cohort.

**Conclusion:** The nomogram provided individualized prognostic quantification of OS in patients with LELCB. BST could yield favorable outcomes when treating LELCB, especially for younger patients, whereas older patients might derive more survival benefit from RC.

## Introduction

The most common histological subtype of epithelial malignancy in the urinary bladder is urothelial carcinoma, with a remarkable propensity for divergent differentiation (1). Compared with the conventional urothelial carcinoma, most variant subtypes were associated with dismal prognosis and recommended to be treated using more aggressive management strategies (2–4). Undifferentiated carcinoma in the nasopharynx with a dense lymphoid infiltrate is denoted as lymphoepithelioma (5). Lymphoepithelioma-like carcinoma (LELC) of the urinary bladder (LELCB) is a relatively rare histological variant, which was first reported by Zukerberg, and is present in 0.4 to 1.3% of all bladder tumors (6, 7). LELCB shows carcinomatous components contrasting with lymphocyte infiltration and mimics chronic inflammation or malignant lymphoma (8). Immunohistochemical staining for cytokeratin helps in the differential diagnosis (9–11). Our incomplete understanding about this enigmatic disease simply comes from small series with a heterogeneous population due to the rare nature of this disease (4). The lack of clinical practice precluded the decision-making process for optimal treatment. Previous studies revealed a favorable outcome of LELCB compared with that of conventional urothelial carcinoma and most other histological variants (4). However, whether it is appropriate to treat muscle-invasive LELCB with bladder-sparing surgery (BSS), including transurethral resection of bladder tumor (TURBT) or partial cystectomy, followed by adjuvant treatment, remains controversial (12–15). In the light of previous experience, effective bladder-sparing therapy (BST) for muscle-invasive bladder cancer requires a judicious selection of patients based on prognostic profiles and risk prediction models. Therefore, the present study was conducted to clarify the prognostic factors of LELCB and design a risk prediction model to assist the decision-making process when treating patients with LELCB.

## Materials and Methods

### Case Sources

The Surveillance, Epidemiology, and End Results (SEER) public-access database covering around 27.8% of the U.S. population was searched from 1973 to 2018, and patients diagnosed with primary urinary bladder cancer [International Classification of Disease for Oncology, 3rd edition (ICD-O-3), primary site: C67.0–C67.9] were identified. The morphology selection was confined to LELC, which was coded as 8082/3 according to the ICD-O-3 criteria. Literature review (LR) was conducted according to the Preferred Reporting Items for Systematic Reviews and Meta-Analyses statement. The established Population, Interventions, Comparators, Outcomes, and Study design (PICOS) strategy was utilized to develop an appropriate search strategy for studies about prognosis and surgical treatment for LELCB patients. The PICOS strategy was defined, in which (P) refers to pathologically diagnosed LELCB patients. Abbreviation (I) corresponds to surgical treatment; (C) non-essential; (O) refers to overall survival defined as time from diagnosis to any cause of death or the last follow-up (up to 120 months); and (S) indicates any study contain information mentioned above. LR was performed online in PubMed and Embase using the following search term: (“lymphoepithelioma-like carcinoma” OR “lymphoepithelioma-like variant” OR “lymphoepithelioma”) AND (“bladder carcinoma” OR “bladder cancer” OR “urinary bladder tumor”). Case reports and case series published before August 5, 2021 were reviewed. Studies lacking information about prognosis, age, sex, evaluation of lymph node involvement, or surgery type were excluded. Language was limited to English only.

### Study Parameters

Parameters of interest included age, gender, tumor extent, lymph node involvement (yes or no), chemotherapy (yes or no), radiation therapy (yes or no), and surgical resection approaches (BSS, RC, or no surgical intervention). In terms of surgical approaches, partial cystectomy and local tumor destruction or excision were categorized as BSS and no surgical treatment as NS. Tumor extent was defined as either non-muscle invasive bladder cancer (NMIBC), or muscle invasive bladder cancer (MIBC) and metastatic bladder cancer (mBC) according to reassigned stage.

### Statistical Analysis

The duration of overall survival (OS) was defined as the time from diagnosis to any cause of death or the last follow-up (up to 120 months). Patients alive at the last follow-up were censored. The Cox proportional hazards regression models were built by the backward stepwise selection method and fitted based on the Akaike Inclusion Criterion in the SEER cohort. Conditional inference tree (CIT), a non-parametric class of regression trees using the permutation tests and multiple test procedures, was applied to classify patients into different prognostic groups using factors identified by the Cox model. The Kaplan–Meier survival curves and log-rank tests were utilized to compare the survival of each prognosis group in the SEER and LR cohorts, and in the subgroup analysis.

The baseline characteristics of patients in the two cohorts were compared. When analyzing differences in categorical variables, the Fisher's exact test was utilized when the sample size at every level was >5; otherwise, the Pearson's chi-squared test was applied. The Mann–Whitney *U*-test was used for continuous variables. All tests were two sided with a statistical significance set at *p* < 0.05. Statistical analyses were performed using R version 3.5.2 (the R Foundation for Statistical Computing, Vienna, Austria).

## Results

### Patient Characteristics

A total of 60 patients diagnosed with LELCB with a median age of 69.5 (interquartile range: 61–75 years) were identified in the SEER database from 1999 to December 2015. LR was performed, and 95 cases from 28 published articles in English from 1993 to 2021 were extracted to verify the performance of prognostic classification derived from the SEER cohort (Supplementary Figure 1) (2, 9, 10, 12–14, 16–32). The baseline characteristics, including age, gender, radiation therapy, tumor extent, and surgery approaches, were statistically similar in both data sets, except for the administration of chemotherapy (Table 1).

**Table 1:** Baseline characteristics of patients with LELCB in the SEER database and from literature review.

**Variables**	**SEER cohort** **(*n* = 60)**	**LR cohort** **(*n* = 95)**	***W*** **/** ***X*** ^**2**^	***p*** **-value**
Age^*^	69.5 (61–75)	71 (65–75)	3091.5	0.736
Sex			0.220	0.639
Male	40	68		
Female	20	27		
Chemotherapy			8.653	0.003
Yes	32	27		
No	28	68		
Radiotherapy			2.492	0.114
Yes	5	18		
No	55	77		
NMIBC			3.306	0.070
Yes	11	7		
No	49	88		
Lymph node involvement			0	1.000
Yes	9	13		
No	51	82		
Surgery^#^			–	0.540
Radical cystectomy	28	42		
Bladder-sparing surgery	29	51		
No surgery	3	2		
Follow-up (months)^*^	29.5 (11–83)	26.0 (12–51)	2606.5	0.372

*LELCB, lymphoepithelioma-like carcinoma of the urinary bladder; LR, literature review; NMIBC, non-muscle invasive bladder cancer; SEER, Surveillance, Epidemiology, and End Results*.

* *Median values are listed with interquartile range in parentheses*.

# *Fisher's exact test was used to test the independence of data from the two cohorts*.

### Prognostic Factors Identified by the Multivariate Cox Model

**Figure 1:**
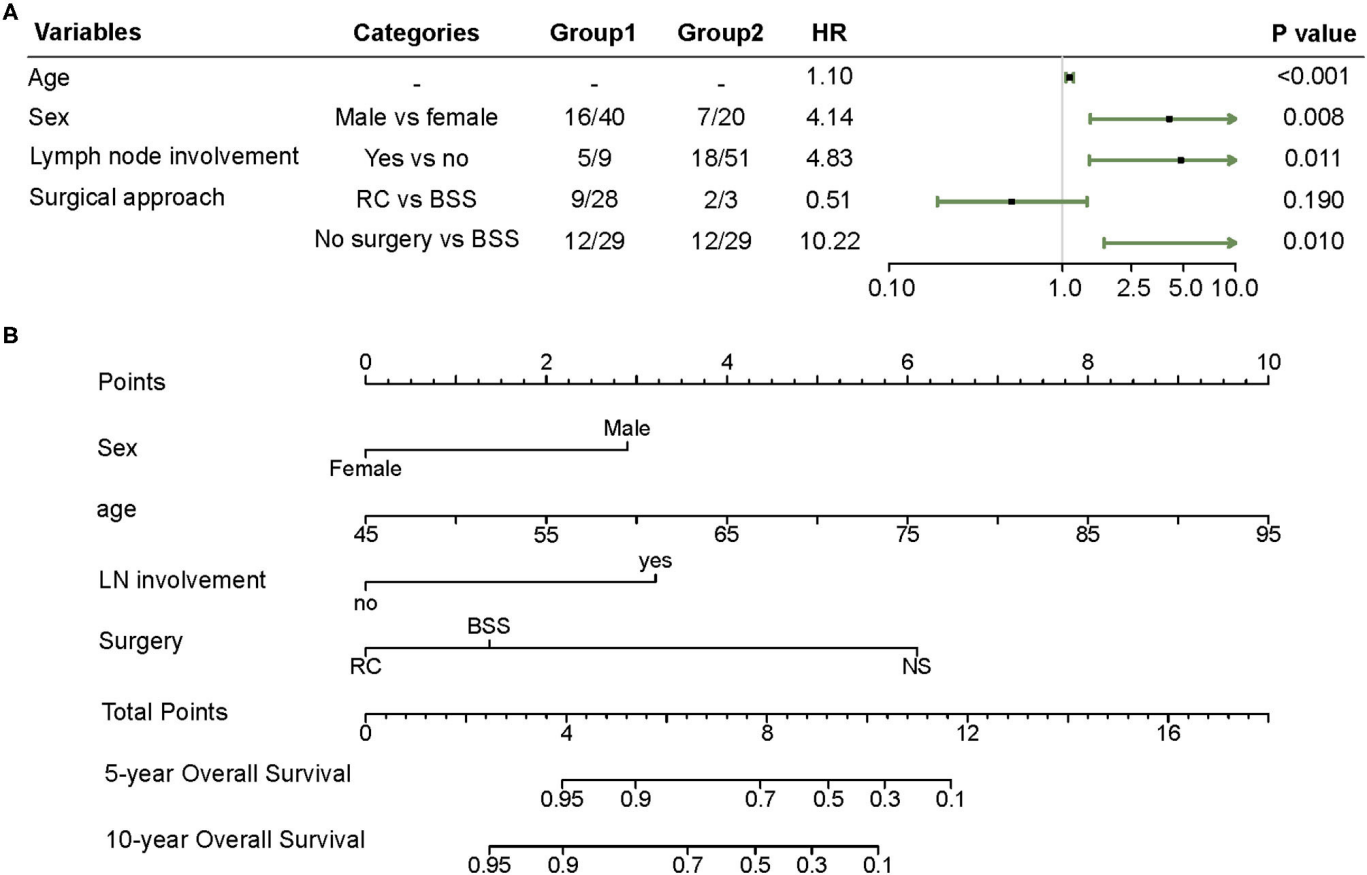
**(A)** Forest plot of the multivariate Cox regression model obtained from the SEER cohort. **(B)** Nomogram predicting 5 and 10 year overall survival (OS) for patients with LELCB. Each variable value is assigned a point and the sum of points can be translated to predict the probability of OS by a line drawn downward on the probability axis. BSS, bladder-sparing surgery; LELCB, lymphoepithelioma-like carcinoma of the urinary bladder; LN, lymph node; NS, no surgery; RC, radical cystectomy.

Variables, such as age, sex, lymph node involvement, and surgery approach, were included in the Cox regression model by the stepwise backward selection method. Male sex (hazard ratio (HR) 4.14, 95% CI 1.45–11.87), metastasis in lymph nodes (HR 4.83, 95% CI 1.43–16.37), and absence of surgical management (HR 10.22, 95% CI 1.74–60.00) showed a strong association with shorter OS (Figure 1A). In the nomogram, the risk point of prognostic factors was assigned according to their contributions to the Cox model as shown in Figure 1B. Furthermore, the OS probability of individual patients was estimated by the total points calculated from the nomogram.

### Comparison of OS Between Prognostic Groups

**Figure 2:**
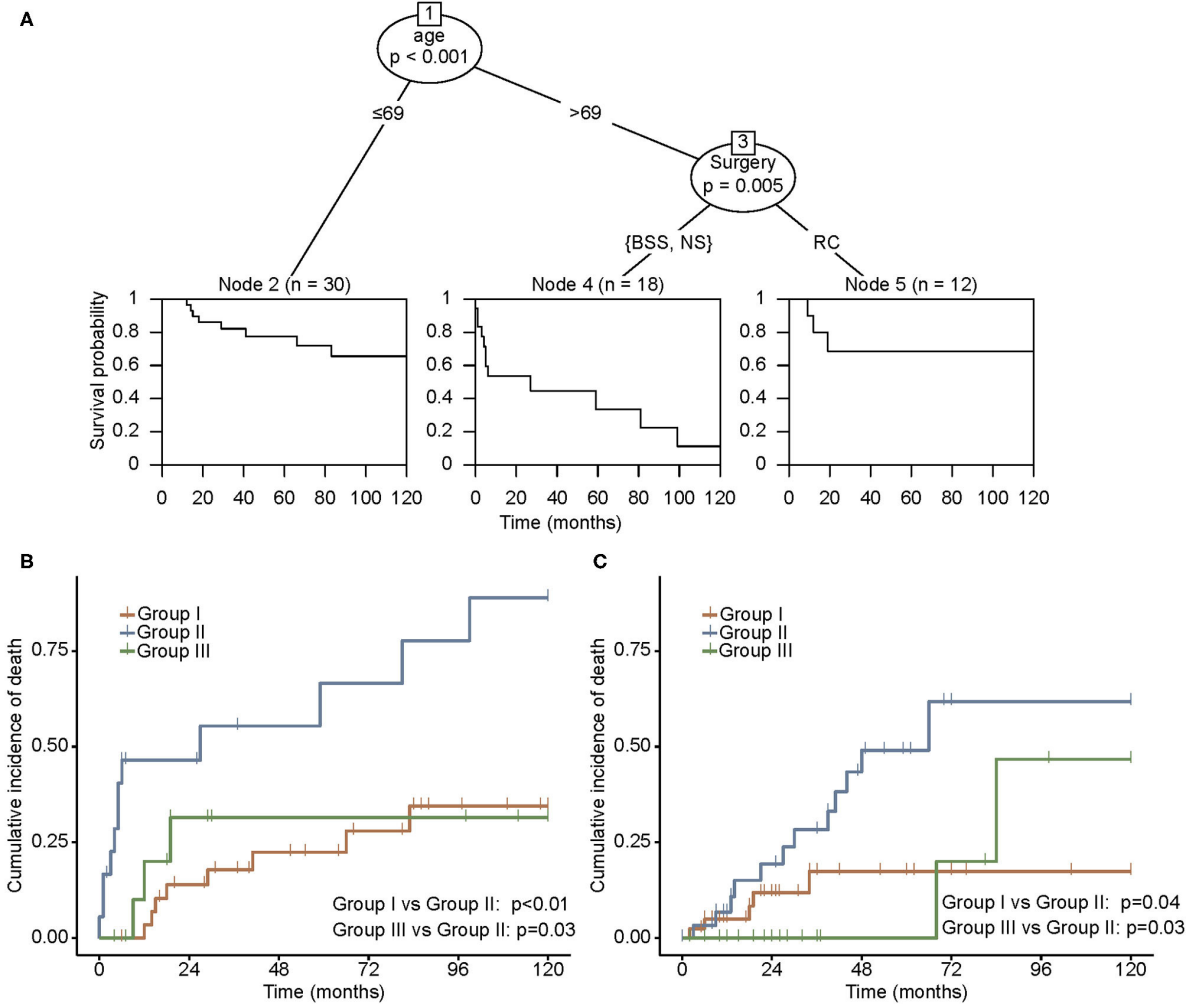
**(A)** Conditional inference tree (CIT) analysis according to the effect of the nomogram score on OS in the SEER cohort. Cumulative incidence of mortality in the SEER cohort **(B)** and the LR cohort **(C)**. Patients were grouped by prognostic classification obtained from CIT. Group I, patients younger than 69 years; group II, patients older than 69 years, receiving BSS or no surgical management; and group III, patients older than 69 years, undergoing RC. BSS, bladder-sparing surgery; LR, literature review; NS, no surgery; OS, overall survival; RC, radical cystectomy; SEER, Surveillance, Epidemiology, and End Results.

In the CIT analysis, age stratified with the cutoff point of 69 years and surgical approach were used to classify patients into three prognostic subgroups (Figure 2A). Patients younger than 69 years were denoted as group I. For patients older than 69 years, those undergoing BSS or no surgical management were sorted into group II, and those who received RC were sorted into group III. The survival functions depicted in each group revealed a remarkable difference in the prognosis.

**Figure 3:**
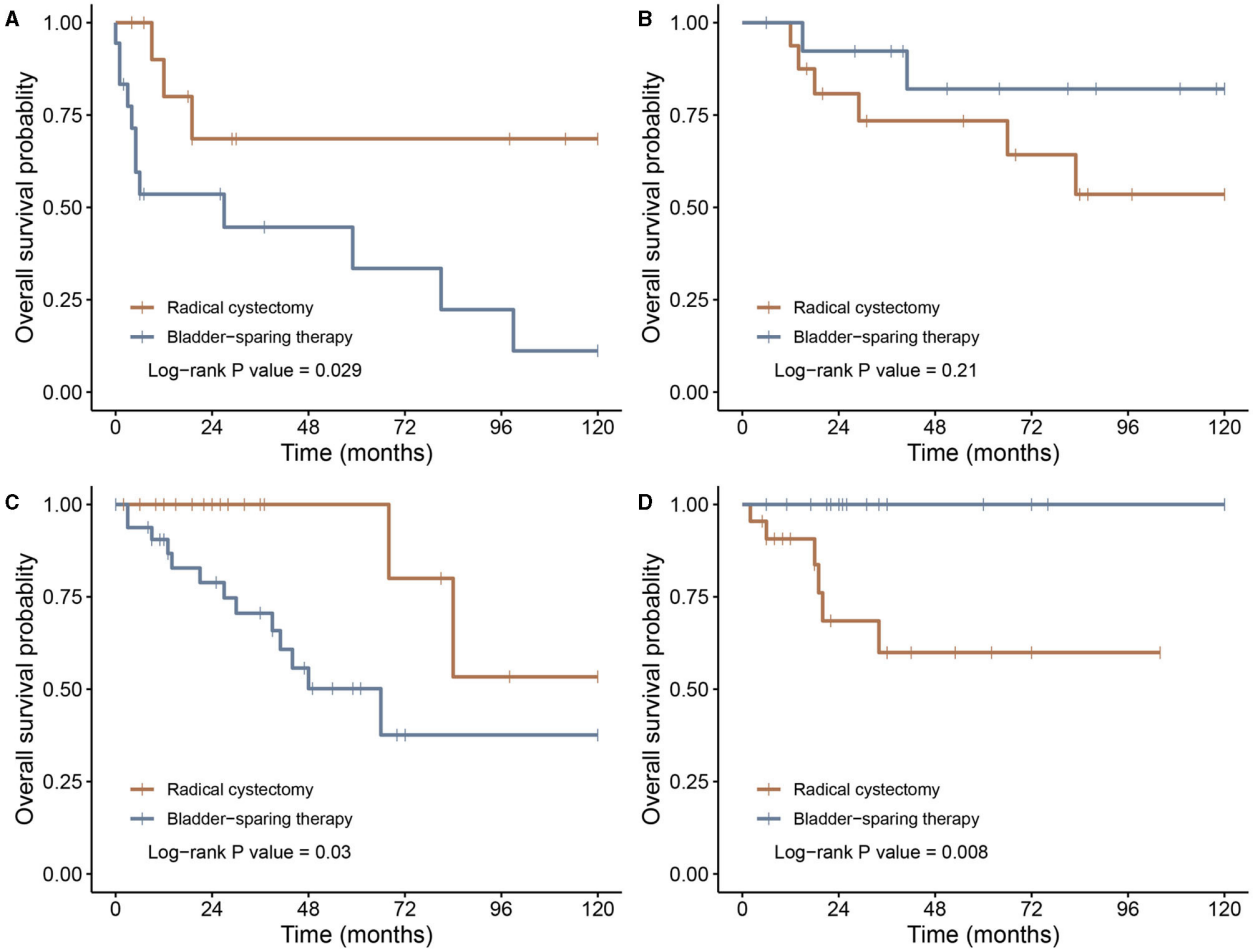
Kaplan–Meier survival curves of patients undergoing radical cystectomy or bladder-sparing therapy. **(A)** Patients older than 69 years in the SEER cohort; **(B)** patients younger than 69 years in the SEER cohort; **(C)** patients older than 69 years in the LR cohort; and **(D)** patients younger than 69 years in the LR cohort. LR, literature review; SEER, Surveillance, Epidemiology, and End Results.

Patients in group II experienced a distinct cumulative incidence of death in the SEER cohort (Figure 2B). A higher incidence of mortality was observed in group II compared with groups I and III (*p* < 0.01 and *p* = 0.03, respectively). The same survival pattern was observed in the LR cohort, where patients in group II possessed higher rates of mortality compared with those in groups I and III (*p* = 0.04 and *p* = 0.03, respectively; Figure 2C). Furthermore, the influence of RC on oncological outcomes for younger and older patients was compared separately. In both SEER and LR cohorts, RC correlated with better survival in the older population, whereas younger patients failed to derive more survival benefits through RC than through BST (Figure 3).

## Discussion

The findings of this study showed that besides age and gender, lymph node metastasis and surgical approach were independent prognostic factors for LELCB. Although LELCB possesses the features of biologically aggressive cancers, its prognosis is favorable compared with most of the other histological variants due to its relatively low probability of metastasis at diagnosis (3, 7, 10, 12, 15). Once tumor cells were detected in lymph nodes, the risk of mortality was 4.83 times higher than those without lymph node involvement in the present study.

Despite its vague intrinsic biological behavior, LELCB was thought to be one kind of urinary bladder cancer that can be properly treated without performing RC on patients with muscle-invasive diseases. Increasing evidence suggested that conservative surgeries combined with chemoradiotherapy could be an alternative to RC (15). Such conclusion was in concordance with our findings that compared with BSS, RC was not an independent prognostic factor in general according to the multivariate Cox model. However, we found that RC could significantly improve survival for patients older than 69 years by taking advantage of the simplicity and comprehensibility of decision trees. Furthermore, RC had no correction with better OS in patients younger than 69 years, indicating that for younger patients, BSS might be an effective alternative.

The majority of LELCB were poorly differentiated or undifferentiated. However, the prognosis of LELCB was superior to that of other histologic variants such as SCC and AC and similar to that of conventional urothelial carcinoma, implying that LELCB was a biologically unique variant and patients should be individualized by different treatment strategies for maximum survival benefit. Some authors hypothesized that such favorable prognosis might be related to an active host response against tumor cells by the predominant lymphoid infiltrate, early symptoms allowing early detection resulting from inflammatory response, and a lower chance of presenting lymph node involvement or distant metastasis at the time of diagnosis (10, 12). Furthermore, it was assumed that higher sensitivity to chemotherapy of LELCB might contribute to such favorable outcomes, leading to the possibility of multimodality treatment to salvage bladder function (8, 10).

A stratification of prognostic value was proposed based on the proportion of LELC component in tumor: pure (100%), predominant (50–99%), and focal (<50%) (12). Despite the lack of such categorization in the SEER database, we rationally assumed that the SEER cohort was comparable to the literature data set because prognostic factors and OS were similar between the two groups. According to a previous review (14), a mass of LELCB of pure and predominant subtypes were successfully treated with TURBT or partial cystectomy, followed by adjuvant therapy, ending up with no evidence of recurrence or progression (33). Furthermore, Yang et al. (15) found a higher rate of no evidence of disease and a significantly lower rate due to death of disease in patients receiving TURBT combined with radiotherapy or chemotherapy after pooling 140 cases from published literature (15). The present study provides insight into the effectiveness of the conservative treatment strategy based on maximal TURBT or partial cystectomy, especially for young patients.

In the current clinical practice, multimodality treatment aiming at functional bladder preservation is adopted in highly selective patients who have T2 tumors without carcinoma *in situ*. Bladder preservation treatment in most circumstances comprises systemic chemotherapy. A disparity in chemotherapy between two datasets was observed in the present study. We deemed this difference acceptable, considering that the prognostic variable selection procedure excluded chemotherapy. A previous study demonstrated that patients receiving combined therapy based on TURBT had comparable disease-free survival of 71.1% compared with patients undergoing RC with disease-free survival of 67.8% (15). Chemotherapy might play an indispensable role in BST for advanced LELCB. Hence, the value of chemotherapy for LELCB in both neoadjuvant and adjuvant settings is worth further exploration.

The present study had several limitations. First, other confounding factors likely to affect survival were not available, such as performance status and molecular features, which are in predicting the biological development of malignancy and the efficacy of targeted therapy or immunotherapy. Second, we were unable to analyze other survival information such as disease-free survival and progression-free survival. Furthermore, a small sample size due to the rare nature of LELCB might lead to potential bias during the statistical analysis. Finally, chemotherapy was not identified as a prognosticator in this study. However, insufficient information on chemotherapy regimen and responses might overshadow the findings of this study, considering that the existing evidence suggesting the use of platinum-based chemotherapy perioperatively might have the potential to improve outcomes (9, 10, 14).

Currently, a partial understanding of the biological behavior of LELCB and difficulty in pathological diagnosis make it challenging to determine the optimal treatment strategy and preclude making individualized management for patients. The present study contributes to the scarce data of LELCB and suggests that BST based on TURBT or partial cystectomy probably yields favorable outcomes for infiltrative LELCB for younger patients, whereas older patients might derive more survival benefits from RC.

## Data Availability Statement

The original contributions presented in the study are included in the article/Supplementary Material, further inquiries can be directed to the corresponding author/s.

## Author Contributions

JW: data collection, data analysis, and manuscript writing. J-ZS: project development. Y-CW: data analysis and manuscript editing. All authors contributed to the article and approved the submitted version.

## Conflict of Interest

The authors declare that the research was conducted in the absence of any commercial or financial relationships that could be construed as a potential conflict of interest.

## Publisher's Note

All claims expressed in this article are solely those of the authors and do not necessarily represent those of their affiliated organizations, or those of the publisher, the editors and the reviewers. Any product that may be evaluated in this article, or claim that may be made by its manufacturer, is not guaranteed or endorsed by the publisher.
