# Supplementary figures and images for: Survival Analysis of Lymphoepithelioma-Like Carcinoma of the Urinary Bladder and the Effect of Surgical Treatment Modalities on Prognosis
Source: Front Surg. 2021 Oct 7;8:706537. doi: 10.3389/fsurg.2021.706537 (PMC8529967; doi:10.3389/fsurg.2021.706537)

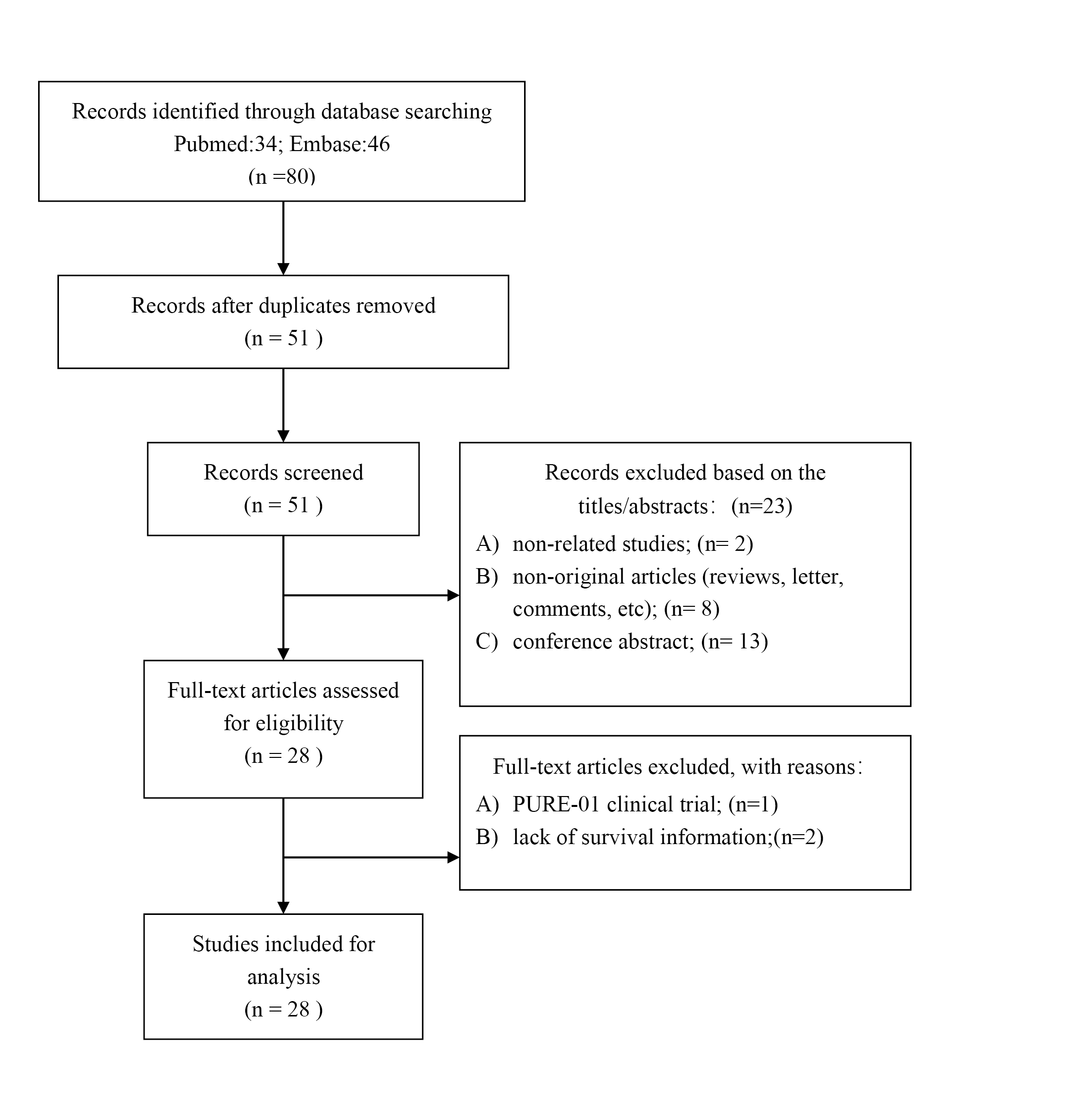

Supplement: Supplementary file 1 [file Image_1.TIF]
